# Evaluation of the Overall Accuracy of the Combined Early Warning Scoring Systems in the Prediction of In-Hospital Mortality

**DOI:** 10.7759/cureus.24486

**Published:** 2022-04-25

**Authors:** Mishal T P, Deepak T S, Aruna C Ramesh, Vikas K N, Thejeswini Mahadevaiah

**Affiliations:** 1 Emergency Medicine, Mathikere Sampige (MS) Ramaiah Medical College, Bengaluru, IND; 2 Anesthesiology, Mathikere Sampige (MS) Ramaiah Medical College, Bengaluru, IND

**Keywords:** intensive care units, survival analysis, triage early warning score, leed’s early warning score, inhospital mortality, early warning scoring systems

## Abstract

Introduction

Deterioration of clinical condition of in-hospital patients further leads to intensive care unit (ICU) transfer or death which can be reduced by the use of prediction tools. The early warning scoring (EWS) system is a prediction tool used in monitoring medical patients in hospitals, hospital staying length, and inpatient mortality. The present study evaluated four different EWS systems for the prediction of patient survival.

Method

The present prospective observational study has analyzed 217 patients visiting the emergency department from November 2016 to November 2018, followed by demographic and clinical data collection. Modified Early Warning Score (MEWS), Triage Early Warning Score (TEWS), Leed’s Early Warning Score (LEWS), and patient-at-risk scores (PARS) were assigned based upon body temperature, consciousness level, heart rate, blood pressure, respiratory rate, mobility, etc. Data was analyzed with the help of R 4.0.4 (R Foundation, Vienna, Austria) and Microsoft Excel (Microsoft, Redmond, Washington).

Results

Out of these 217 patients, 205 got shifted to a ward, and 12 died, amongst which the majority belonged to the 31-40 age group. Among patients admitted to ICU had a MEWS greater than 3, TEWS within the range 0 to 2 and 3 to 5, LEWS greater than 7, and PARS greater than 5 on the initial days of admission. The patients who died and those who were shifted to the ward showed significant differences in EWS. A significant association was observed between all the EWS and patient outcomes (p<0.001).

Conclusion

MEWS, TEWS, LEWS, and PARS were effective in the prediction of inpatient mortality as well as admission to the ICU. With the increase in the EWS, there was an increase in the duration of ICU stay and a decrease in chances of survival.

## Introduction

Different patients face unanticipated clinical collapse during hospitalization, which can further lead to in-hospital mortality [1-3]. There are several reports which show that the majority of patients manifest signs of high risk much before their health actually starts to deteriorate. These preliminary signs can be used to predict patients at risk by monitoring and analyzing the measured clinical data. The main focus behind such prediction is the improvement of patient prognosis. The different rationale has been adapted for developing scoring systems for predicting in-hospital mortality with the help of a good triage tool [4,5]. In this respect, the use of this scoring system during caring for these patients in the emergency department (ED) can also delay treatment of these patients in whom mortality calculation has been done or for the detection of deficiencies that become prominent [6]. The simplicity as well as ease in the calculation of these scoring systems has a high power in mortality estimation, and helps the clinician to be more careful [7]. These early warning scores employ different factors like vital signs as well as laboratory results in addition to subjective parameters, which are considered as input to generate an output in the form of an integer score. A higher score might be an indication of the probability to undergo clinical collapse but is not a direct measure in any way [8].

The usefulness of early warning systems (EWS) has increased drastically in acute care units of hospitals in different countries like the Netherlands, USA, UK, Denmark, and South Korea [9,10]. Various EWS like Modified Early Warning Scoring System (MEWS), Triage Early Warning Scoring (TEWS), Leeds Early Warning Scoring (LEWS), patient-at-risk scoring (PARS), etc. have been developed with the aim of identifying the patients at risk at an early stage so that proper measures can be taken for saving the patient [11]. EWS was defined by Morgan et al. in the year 1997 [12]. Stenhouse et al. further modified this early warning scoring system and defined the MEWS calculated by measurement of pulse rate, respiratory rate, fever temperature, consciousness levels, and systolic blood pressure along with urinary excretion [13]. TEWS is another useful tool that has been exploited for identifying patients in the ED who are at risk of deterioration and would further require ICU admission [14]. There is another scoring system which has been developed by Leeds University teaching hospitals in 2001 called the LEWS, which aimed at improving the straightforward approach of MEWS along with retention of its sensitive nature [15]. Recently, a slightly different scoring system was developed by Goldhill et al., which was called the patient-at-risk score (PARS), with different scores for similar observations in addition to the included urinary excretions [16]. However, the efficiency of the EWS in identifications of patients with the risk of in-hospital mortality during admission has not been validated using different scoring systems due to associated risk factors, thereby influencing the system.

Here, we have assessed the use of four different early warning scoring systems, Modified Early Warning Score (MEWS), Triage Early Warning Score (TEWS), Leed’s Early Warning Score (LEWS), and Patient-at-Risk Score (PARS), in adult patients triaged using emergency severity index. Triage scores were calculated from patients that were admitted to the medical intensive care unit (MICU) and further used for the determination of survival prediction.

## Materials and methods

A total of 217 patients who were admitted to the intensive care unit of the emergency department of MS Ramaiah medical college hospital in Bangalore during the period from November 2016 to November 2018 have been evaluated in this prospective observational study. Written informed consent was obtained from the patients, after which patient information was obtained from medical records. The study was issued ethical clearance by the Institutional Ethics Committee (ECR/215/Inst/Ker/2013).

Data collection

All patients who were admitted to the emergency department and triaged using the emergency service index (ESI) triage scoring system were included in the study [17]. However, patients not requiring intensive care unit admission, violent patient attenders, and patients/attendants not giving consent for the study were excluded. After assessment and admission to ICU, data collection was done for these patients, which includes details regarding age, sex, date of admission, co-morbidities, diagnosis, day-wise scoring using different scoring systems as well as patient outcome. MERS, TEWS, LEWS, and PARS were assigned based upon body temperature, consciousness level, heart rate, blood pressure, respiratory rate, mobility, etc. In the present study, we expected a combined sensitivity of 83% by LEWS, MEWS, PARS, and TEWS scores, and to get 5% absolute precision with a 95% confidence level in the result, the study required a minimum of 217 subjects.

Data was analyzed using the software R version 4.0.4 (R Foundation, Vienna, Austria) and Microsoft Excel (Microsoft, Redmond, Washington). Continuous and categorical variables were presented as a frequency and mean ± SD/median (min, max) respectively. Two-sample t-test/ Mann Whitney U test was used to compare the means/distributions of variables. The Chi-square test is used to check the association between attributes. A p-value of </= 0.05 indicates statistical significance.

## Results

The present study was performed using 217 patients whose age ranges from 17 years to 92 years, with a mean age of 51.67 ± 20.09 years. The baseline characteristics presented by the patient population have been represented in Table 1. Of these 217 patients included in this study, 52.07% of patients were males, and the rest, 47.93%, patients were females. There were 12 patients (5.5%) who died during hospitalization, whereas the other 205 patients (94.5%) were shifted to the wards. There were a large number of patients within the age groups 31-40 (17.1%) and 61-70 (17.1%), whereas only one patient (0.5%) was in the age group above 90 years. Different co-morbidities such as hypertension (HTN) (41.67%), type 2 diabetes mellitus (T2DM) (50%), coronary artery disease (CAD) (33.33%), and chronic kidney disease (CKD) (8.33%) were observed in expired patients.

**Table 1:** Baseline characteristics of the patient population HTN - hypertension, T2DM - type 2 diabetes mellitus, CAD - coronary artery disease, CKC - chronic kidney disease, C - Chi-square test, MC - Chi-square test with Monte Carlo simulation, t - Two-sample t-test, * indicates statistical significance.

Variables	Sub-category	Outcome		Total	p-value
		Expired 12 (5.53%)	Shifted to Ward 205 (94.47%)		
Age (years)	≤20	0	11 (5.37%)	11 (5.07%)	0.949^MC^
	21-30	2 (16.67%)	26 (12.68%)	28 (12.9%)	
	31-40	3 (25%)	35 (17.07%)	38 (17.51%)	
	41-50	2 (16.67%)	30 (14.63%)	32 (14.75%)	
	51-60	2 (16.67%)	27 (13.17%)	29 (13.36%)	
	61-70	2 (16.67%)	35 (17.07%)	37 (17.05%)	
	71-80	1 (8.33%)	20 (9.76%)	21 (9.68%)	
	81-90	0	20 (9.76%)	20 (9.22%)	
	>90	0	1 (0.49%)	1 (0.46%)	
	Mean ± SD	47.33 ± 17.27	51.92 ± 20.25	51.67 ± 20.09	0.4432^t^
Gender	Male	6 (50%)	107 (52.2%)	113 (52.07%)	0.8824^C^
	Female	6 (50%)	98 (47.8%)	104 (47.93%)	
HTN	No	7 (58.33%)	119 (58.05%)	126 (58.06%)	0.9845^C^
	Yes	5 (41.67%)	86 (41.95%)	91 (41.94%)	
T2DM	No	6 (50%)	120 (58.54%)	126 (58.06%)	0.5602^C^
	Yes	6 (50%)	85 (41.46%)	91 (41.94%)	
CAD	No	8 (66.67%)	184 (89.76%)	192 (88.48%)	0.04198^MC^*
	Yes	4 (33.33%)	21 (10.24%)	25 (11.52%)	
CKD	No	11 (91.67%)	182 (88.78%)	193 (88.94%)	1^MC^
	Yes	1 (8.33%)	23 (11.22%)	24 (11.06%)	

Statistical analysis was carried out to test the correlation between the associated variables like age, gender, and co-morbidities with patient outcomes using the Chi-square test, Chi-square test with Monte Carlo simulation as well a two-sample t-test. The data revealed that there was no significant difference in mean age in subjects with patient outcomes. Moreover, there was no significant association of age, gender as well as co-morbidities like HTN, T2DM, and CKD with patient outcomes. However, CAD had significantly associated with patient outcomes (p=0.04198).

Different scoring systems like MEWS, TEWS, LEWS, and PARS have been used for prediction in patients at the risk of deterioration. Summaries of MEWS, TEWS, LEWS, and PARS up to day eight in this study have been represented in Table 2, Table 3, Table 4, and Table 5, respectively. Although the scoring was performed for the next 10 days, the results were insignificant on day nine and day 10, therefore it is not mentioned in Table 2, Table 3, Table 4, and Table 5. 56.22% of the patients admitted to the ICU had MEWS greater than three and the remaining 43.78% had MEWS less than three on day one (Table 2). The majority of the patients admitted to the ICU had TEWS between 0-2 (45.62%) and 3-5 (41.94%) on the first day (Table 3). There were 71.43% of patients who were admitted to the ICU with LEWS less than seven, and the remaining 28.57% of patients had LEWS greater than seven on day one (Table 4). 38.89% of the patients admitted to the ICU had PARS between 0-2, 34.72% had PARS between three to five, and 26.39% had PARS greater than five on day one (Table 5).

**Table 2:** Distribution of Modified Early Warning Scores (MEWS) with respect to duration of admission

Variable	Timepoint	No. of patients N	Scores, mean ± SD	Scores, median (min, max)	Scores <3	Scores ≥3
MEWS	Day 1	217	3.56 ± 2.64	3 (0, 16)	95 (43.78%)	122 (56.22%)
	Day 2	212	2.47 ± 1.6	2 (0, 11)	131 (61.79%)	81 (38.21%)
	Day 3	193	1.69 ± 1.27	1 (0, 10)	169 (87.56%)	24 (12.44%)
	Day 4	85	1.54 ± 1.01	1 (0, 8)	76 (89.41%)	9 (10.59%)
	Day 5	29	1.83 ± 1.14	1 (1, 5)	22 (75.86%)	7 (24.14%)
	Day 6	16	1.88 ± 1.02	2 (1, 5)	14 (87.5%)	2 (12.5%)
	Day 7	8	1.62 ± 0.74	1.5 (1, 3)	7 (87.5%)	1 (12.5%)
	Day 8	1	1	1 (1, 1)	1 (100%)	-

**Table 3:** Distribution of Triage Early Warning Scores (TEWS) with respect to duration of admission

Variable	Timepoint	No. of patients N	Scores, mean ± SD	Scores, median (min, max)	Scores 0-2	Scores 3-5	Scores 6-7	Scores >7
TEWS	Day 1	217	3.28 ± 2.25	3 (1, 15)	99 (45.62%)	91 (41.94%)	18 (8.29%)	9 (4.15%)
	Day 2	212	2.46 ± 1.6	2 (0, 11)	135 (63.68%)	66 (31.13%)	7 (3.3%)	4 (1.89%)
	Day 3	193	1.71 ± 1.29	1 (0, 12)	167 (86.53%)	23 (11.92%)	1 (0.52%)	2 (1.04%)
	Day 4	85	1.62 ± 0.95	1 (1, 6)	74 (87.06%)	10 (11.76%)	1 (1.18%)	-
	Day 5	29	1.97 ± 1.18	2 (1, 5)	21 (72.41%)	8 (27.59%)	-	-
	Day 6	16	1.94 ± 0.77	2 (1, 3)	12 (75%)	4 (25%)	-	-
	Day 7	8	1.88 ± 0.83	2 (1, 3)	6 (75%)	2 (25%)	-	-
	Day 8	1	1	1 (1, 1)	1 (100%)	-	-	-

**Table 4:** Distribution of Leed’s Early Warning Scores (LEWS) with respect to duration of admission

Variable	Timepoint	No. of patients N	Scores, mean ± SD	Scores, median (min, max)	Scores <7	Scores ≥7
LEWS	Day 1	217	5.28 ± 3.83	4 (0, 19)	155 (71.43%)	62 (28.57%)
	Day 2	212	3.67 ± 2.61	3 (0, 15)	182 (85.85%)	30 (14.15%)
	Day 3	193	2.33 ± 2.01	2 (0, 14)	185 (95.85%)	8 (4.15%)
	Day 4	85	2.19 ± 1.77	2 (0, 12)	82 (96.47%)	3 (3.53%)
	Day 5	29	2.66 ± 2.02	3 (0, 8)	26 (89.66%)	3 (10.34%)
	Day 6	16	2.81 ± 1.87	3 (1, 7)	15 (93.75%)	1 (6.25%)
	Day 7	8	2.62 ± 1.6	2.5 (1, 5)	8 (100%)	-
	Day 8	1	2	2 (2, 2)	1 (100%)	-

**Table 5:** Distribution of patient-at-risk scores (PARS) with respect to duration of admission

Variable	Timepoint	Number of patients N	Scores, mean ± SD	Scores, median (min, max)	Scores 0-2	Scores 3-5	Scores >5
PARS	Day 1	217	4.12 ± 3.13	3 (0, 16)	84 (38.89%)	75 (34.72%)	57 (26.39%)
	Day 2	212	2.73 ± 1.95	2 (0, 14)	122 (57.82%)	73 (34.6%)	16 (7.58%)
	Day 3	193	1.78 ± 1.38	1 (0, 10)	161 (83.42%)	29 (15.03%)	3 (1.55%)
	Day 4	85	1.69 ± 1.18	1 (0, 9)	74 (87.06%)	10 (11.76%)	1 (1.18%)
	Day 5	29	2.14 ± 1.36	2 (1, 6)	20 (68.97%)	8 (27.59%)	1 (3.45%)
	Day 6	16	2.19 ± 1.17	2 (1, 5)	11 (68.75%)	5 (31.25%)	-
	Day 7	8	2 ± 1.2	1.5 (1, 4)	5 (62.5%)	3 (37.5%)	-
	Day 8	1	1	1 (1, 1)	1 (100%)	-	-

Statistical analysis was carried out to test the association between the MEWS, TEWS, LEWS, and PARS for the first two days with patient outcomes using the Mann Whitney U test (Table 6). Correlation between different scoring systems and patient outcomes were compared to the values, as shown in Table 6. Mean outcome on day one among all scoring systems ranged between 9.00 ± 2.94 to 12.00 ± 5.46 in patients who expired as compared to 2.29 ± 1.24 to 4.93 ± 3.35 in patients who got shifted to a ward. Complete details of each scoring system are mentioned in Table 6. MEWS, TEWS, LEWS, and PARS showed a positive correlation with patient outcomes (p<0.001), as represented in Table 6.

**Table 6:** Correlation between different scoring systems and patient outcomes MEWS - Modified Early Warning Score, TEWS - Triage Early Warning Score, LEWS - Leed’s Early Warning Score, PARS - patient-at-risk score, * indicates statistical significance

Variables	Sub-category	Outcome		Total	p-value
		Expired	Shifted to a ward		
MEWS	Day 1	9.17±3.81	3.27±2.18	3.59±2.65	< 0.001*
	Day 2	7.86±1.95	2.31±1.24	2.50±1.61	< 0.001*
TEWS	Day 1	8.58±3.87	3.00±1.70	3.31±2.26	< 0.001*
	Day 2	8.00±1.63	2.29±1.24	2.48±1.61	< 0.001*
LEWS	Day 1	12.00±5.46	4.93±3.35	5.32±3.84	< 0.001*
	Day 2	10.86±2.04	3.47±2.29	3.71±2.63	< 0.001*
PARS	Day 1	10.58±4.56	3.78±2.60	4.15±3.14	< 0.001*
	Day 2	9.00±2.94	2.55±1.54	2.76±1.97	< 0.001*

## Discussion

Early warning scoring systems have been used as a prediction tool by using clinical signs to estimate the patients’ stay in hospitals [18]. The correlation of EWS with inpatient mortality as well as hospital admission has been largely studied. The higher the score value, the higher the chance of ICU admission into the hospital [19]. Different types of scoring systems such as MEWS, TEWS, LEWS, and PARS, which make use of various physiological parameters for defining a patient’s score, have proved their efficacy in determining deterioration in patients. Thus, EWS enables the hospital staff to recognize patient outcomes, thereby enabling early interventions.

Introducing an Early Warning Scoring (EWS) is complex. Many observational studies, as well as pre-intervention and post-intervention evaluation analyses indicating EWS systems, have improved the detection of deterioration clinically [20,21]. Previous studies have shown that MEWS might be useful in reducing mortality in hospital patients [22]. Thus Modified Early Warning Score (MEWS) will be a protocol to identify patients in hospitals who are at risk of unexpected deterioration catastrophically [23]. Use of TEWS upon patient admission aids in identifying patients at risk of deterioration clinically which was similar to previous reports showing a correlation between TEWS and patient outcomes. The findings of the study are in keeping with those studies which had demonstrated an association between TEWS and the outcome [24]. Similarly, LEWS and PARS also have been observed to be used in the prediction of patient outcomes, thereby reducing inpatient mortality. There are various reports which make use of different scoring systems for calculating risk scores in general inpatients; however, their relative performance is incompletely characterized in the Indian scenario. Hence, a comparative analysis of different scoring systems has been done in the present study for a better understanding of their performances.

The present study has shown an efficient prediction of mortality using EWS with respect to inpatient mortality, increased probability of ICU admission, increase in hospital stay, and ultimately death. The EWS thus has been used as a triage tool in the emergency department for acute medical patients and further identification of patients at risk. The EWS further aids in the identification of vulnerable patients, thereby helping medical staff in making suitable arrangements like bed type (general ward/high dependency), the suitable interval for nursing observations as well as physician review.

In the current study, a significant association was observed between increased TEWS and increased admission to hospital as well as inpatient mortality. A mean TEWS value of 8.58 ± 3.87 was observed in patients who died on the first day as compared to 3.00 ± 1.70 in those who were shifted to the ward. Therefore, TEWS calculation on patients’ admission into the emergency room can be used for identifying patients at risk of clinical deterioration. The results are similar to those findings from other studies which demonstrated the association of TEWS with outcomes that indicated higher TEWS on patient admission as a measure for prediction of the necessity of admission as well as increased mortality [25, 26]. In a similar study, the probability of ICU admission remarkably increased in patients with TEWS of 3-4 (p<0.001) as compared to those with TEWS of 7-14 (p<0.01), thus underlining the utility of TEWS in predicting patient outcomes [27].

The present study has demonstrated a mean LEWS value of 12.00±5.46 on day one in patients who died in comparison to a LEWS value of 4.93 ± 3.35 in those who got shifted to the hospital’s ward. Similar results have been observed in various other studies suggesting a LEWS value of more than seven to be the most precise prediction of in-hospital mortality as well as survival [11].

Subbe et al. and Armagan et al. have inferred that a MEWS value greater than five has been associated with a high probability of ICU admission and death. Also, MEWS values help in identifying the patients at risk of deterioration clinically with the necessity of increased care in the ICU [28, 29]. This was even evident in the present study, where a mean MEWS value of 9.17 ± 3.81 was observed in patients who had expired on day one in comparison with a MEWS value of 3.27 ± 2.18 in those who got shifted to the hospital’s ward.

Similarly, even PARS has been observed to be an efficient prediction tool for assessing an individual patient with the response for the initial resuscitation along with identification of patients who need further assessment by specialist doctors before getting discharged from the hospital [30]. This observational study showed higher mean PARS values of 10.58 ± 4.56 on the first day in patients who expired as compared to mean PARS values of 3.78 ± 2.60 in patients who were shifted to the hospital’s ward.

Limitations

There are some limitations associated with this study, however. Firstly, this was a single center-based prospective observational study with a small sample size, because of which there is a possibility of bias. This bias can be nullified by performing a double-blinded multi-center analysis with a large population size. Secondly, selection bias cannot be ruled out as many patients with missing variables have been excluded. Also, the number of patients coming for regular follow-ups decreased with time duration. Thirdly, more accurate identification of risk patients based on different scoring systems does not essentially improve patient outcomes. Even though these limitations cannot be ignored, the present study does show its clinical importance in highlighting the use of different EWS for predicting patient outcomes.

## Conclusions

EWS is a beneficial tool used for relevant risk management, thereby optimizing the quality and safety of the patients who have been admitted to the emergency department by allowing early intervention, ultimately leading to tremendous improvement in quality of care as well as a decrease in morbidity and mortality. The present study has highlighted the use of different early warnings scores like MEWS, TEWS, LEWS, and PARS for the prediction of patient outcomes. MEWS, TEWS, LEWS, and PARS were effective in the prediction of inpatient mortality as well as admission to the ICU.
